# Comparison of Extruded and Sonicated Vesicles for Planar Bilayer Self-Assembly

**DOI:** 10.3390/ma6083294

**Published:** 2013-08-05

**Authors:** Nam-Joon Cho, Lisa Y. Hwang, Johan J.R. Solandt, Curtis W. Frank

**Affiliations:** 1 School of Materials Science and Engineering, Nanyang Technological University, 50 Nanyang Avenue, 639798, Singapore; 2 Department of Chemical Engineering, Stanford University, Stauffer III, 381 North-South Mall, Stanford, CA 94305, USA; E-Mails: lyhwang@stanford.edu (L.Y.H.); johan.solandt@astrazeneca.com (J.J.R.S.)

**Keywords:** vesicle, liposome, planar bilayer, extrusion, sonication, dynamic light scattering, quartz crystal microbalance, fluorescence recovery after photobleaching, atomic force microscopy, supported lipid membranes

## Abstract

Lipid vesicles are an important class of biomaterials that have a wide range of applications, including drug delivery, cosmetic formulations and model membrane platforms on solid supports. Depending on the application, properties of a vesicle population such as size distribution, charge and permeability need to be optimized. Preparation methods such as mechanical extrusion and sonication play a key role in controlling these properties, and yet the effects of vesicle preparation method on vesicular properties and integrity (e.g., shape, size, distribution and tension) remain incompletely understood. In this study, we prepared vesicles composed of 1-palmitoyl-2-oleoyl-*sn*-glycero-3-phosphocholine (POPC) lipid by either extrusion or sonication, and investigated the effects on vesicle size distribution over time as well as the concomitant effects on the self-assembly of solid-supported planar lipid bilayers. Dynamic light scattering (DLS), quartz crystal microbalance with dissipation (QCM-D) monitoring, fluorescence recovery after photobleaching (FRAP) and atomic force microscopy (AFM) experiments were performed to characterize vesicles in solution as well as their interactions with silicon oxide substrates. Collectively, the data support that sonicated vesicles offer more robust control over the self-assembly of homogenous planar lipid bilayers, whereas extruded vesicles are vulnerable to aging and must be used soon after preparation.

## 1. Introduction

Synthetic lipid vesicles (also referred to as liposomes) are topologically closed assemblies of single or multiple lipid bilayers that are increasingly important biomaterials for a wide range of delivery and formulation applications in the pharmaceutical and cosmetic industries, among other fields [1,2,3]. With liposome-based products commanding multi-billion dollar market opportunities in total, liposome manufacturing is a growing field that has evolved over the last 30 years and is yielding tremendous benefits for application-specific formulations and optimized processing techniques. Homogenous samples and stringent quality control are key target goals of any manufacturing protocol, and a handful of preparation techniques, including sonication and mechanical extrusion, meet these criteria and are thus widely used in academia and industry [4,5,6].

Depending on the application, sonication and extrusion have varying advantages that make them the two of the most common preparation methods for vesicles [7]. Extrusion can produce monodisperse unilamellar vesicle suspensions as a result of energy-dissipating shearing forces that are generated when lipid suspensions are repeatedly passed through polymer membranes containing well-defined, nanoscale pores under high-pressure conditions [8,9]. Although extrusion offers high sample reproducibility, it is also relatively slow, manual and cumbersome to implement. Moreover, some sample material may be lost during extrusion during passage through the porous membrane. By contrast, sonication uses acoustic energy to induce pressure waves that break up large, multilamellar vesicles and aggregates into smaller vesicles. The administration time and the intensity of the pressure waves largely determine the size of the processed vesicles, and in general sonication is quicker and less-intensive than extrusion [10,11].

As the number of applications for vesicles continues to rise, there is a growing need to move beyond size characterization and to fundamentally understand how physical properties such as diffusion and permeability of the liposomal bilayer are affected by the processing technique employed. Control over these properties can be critical in specific applications such as membrane permeability for controlled drug release [12] or lateral receptor mobility for antibacterial decoy therapeutics [13]. To this end, Lapinski *et al.* recently measured the rotational and translational diffusion of vesicles composed of 1-palmitoyl-2-oleoyl-*sn*-glycero-3-phosphocholine (POPC) lipids immediately after sonication or extrusion, and found that these properties are unaffected by the processing technique [14]. This finding led to the proposition that the molecular scale organization of bilayers may not be significantly influenced by preparation method and, therefore, data on vesicles formed by sonication or extrusion may be directly compared if all other parameters are fixed.

Since this conclusion has potentially broad implications for vesicle-based technologies and products, we reason that it is important to further investigate this finding in the context of specific applications. Solid-supported planar lipid bilayers formed by the fusion of vesicles with hydrophilic substrates including silicon oxide [15,16] and mica [17,18] are important cell membrane mimics for biosensing and antifouling coatings, and take advantage of robust self-assembly depending on experimental conditions including vesicle size [19], lipid composition [20], ionic strength [21] and solution pH [22,23,24]. With emerging commercial opportunities for these platforms in pharmaceutical drug discovery and development [25], quality control of these platforms needs to be extended to include industry-relevant parameters such as stability of stored vesicles and processing method employed. Although there have been inconsistent findings in the literature that are possibly attributable to the vesicle preparation method [26,27] (*i.e.*, sonication *vs.* extrusion), the effects of processing step conditions on the self-assembly of solid-supported planar lipid bilayers are not well understood.

To address this gap, we investigate how preparation method and aging of vesicles affect the self-assembly of planar lipid bilayers. By employing a battery of analytical techniques such as dynamic light scattering (DLS), quartz crystal microbalance with dissipation (QCM-D) monitoring, fluorescence recovery after photobleaching (FRAP) and atomic force microscopy (AFM), a quantitative framework is established to correlate vesicle preparation method with the propensity to form homogenous planar lipid bilayers on silicon oxide, as determined by bilayer mass, viscoelasticity, lateral mobility and morphology. We found that while vesicles prepared by sonication formed homogenous planar bilayers through an identical self-assembly pathway regardless of aging conditions, vesicles prepared by mechanical extrusion not only exhibited age-dependent self-assembly interaction kinetics, but the quality of the planar bilayer formed deteriorated as a function of the age of the precursor vesicles. Taken together, the data indicate that the physical properties of planar lipid bilayers can vary depending on properties such as vesicle preparation method and aging. Since sonication and extrusion are the two most common methods to prepare vesicles for solid-supported model membranes, these findings are broadly relevant for academic and industrial research and development. These results offer a caveat that vesicle preparation method might be an important variable depending on the nature of the specific application at hand.

## 2. Materials and Methods

### 2.1. Vesicle Preparation

Small unilamellar vesicles (SUVs) composed of 1-palmitoyl-2-oleoyl-*sn*-glycero-3-phosphocholine (POPC) lipid (Avanti Polar Lipids, Alabaster, AL, USA) were prepared by the extrusion method or sonication. A Tris buffer with the following composition was used for all experiments: 10 mM Tris (pH 7.5), and 150 mM NaCl solution with 1 mM ethylene diamine tetracetic acid (EDTA) in 18.2 MΩ·cm MilliQ water (MilliPore, Billerica, MA, USA). Extruded unilamellar vesicles (referred to simply as vesicles) were prepared in the following manner: lipid films were prepared by first drying the as-supplied lipids dissolved in chloroform under a gentle stream of nitrogen at room temperature. Then, the resulting lipid film was stored under vacuum for at least 5 h in order to remove residual chloroform. Multilamellar vesicles (MLVs) were prepared by next swelling the lipid film in aqueous solution, then vortexing periodically for 5 min. The resulting MLVs were subsequently processed by either extrusion or sonication. For extrusion, MLVs were passed through track-etched polycarbonate membranes with nominal 100-nm pores at least 11 times using a mini-extruder apparatus (Avanti Polar Lipids, Alabaster, AL, USA). For sonication, MLVs were subjected to ultrasound radiation using a Vibracel titanium-tip sonicator (Tecnochimica Sassolese, Modena, Italy) with a maximum power of 600 W and frequency of 20 kHz. Each sample underwent repetitive 3 Hz cycles that consisted of 1 s pulses at a power of 150 W to control thermal effects. In order to remove larger particulates (e.g., lipid aggregates and/or the small fraction of large, multilamellar vesicles typically found in the bimodal distribution) [28,29] and have a comparable size distribution, we extruded the sonicated vesicles one-time through a 100 nm polycarbonate etch-track membrane as a filtration step. Vesicles were generally prepared at a nominal lipid concentration of ~5 mg·mL^−1^, and then diluted to ~0.1 mg·mL^−1^ before experiment. Throughout the study, vesicles were stored in a dark refrigerator at 4 °C.

### 2.2. Dynamic Light Scattering (DLS)

The effective diameter of vesicles was measured using dynamic light scattering with a Brookhaven 90 Plus Particle Analyzer (Brookhaven Instruments Corporation, Holtsville, NY, USA) at 25 °C. The scattering data were analyzed by digital autocorrelator software (Brookhaven Instruments Corporation) [30]. All measurements were taken at a scattering angle of 90° where the reflection effect is minimized. All autocorrelation functions were also analyzed by CONTIN and Non-Negatively Constrained Least Squares (NNLS) algorithms to check for multi-modal distributions.

### 2.3. Quartz Crystal Microbalance with Dissipation (QCM-D)

Adsorption kinetics and the viscoelastic properties of the adsorbed layer were studied using a Q-Sense D300 (Q-Sense AB, Gothenburg, Sweden) equipped with a QWiC 301 window chamber (Q-Sense AB). The crystal was initially driven near its resonance frequency, as indicated by a maximum in the current. To capture the characteristic dissipation, the drive circuit was short-circuited and the exponential decay of the crystal oscillation was recorded and analyzed, yielding the frequency and dissipation changes at 5, 15, 25 and 35 MHz. The temperature of the measurement cell was set at 25.0 °C and accurately controlled by a Peltier element in the cell with fluctuations smaller than ±0.05 °C. AT-cut crystals (Q-Sense AB) of 14 mm in diameter with 50 nm thermally evaporated SiO_x_ were used for all experiments. Each QCM crystal was treated with oxygen plasma at ~80 watts for ~5 min prior to use (March Plasmod Plasma Etcher, March Instruments, Concord, CA, USA).

### 2.4. Fluorescence Recovery after Photobleaching (FRAP)

Fluorescence recovery after photobleaching (FRAP) measurements were performed on mica-supported planar lipid bilayers using an Eclipse E800 upright microscope (Nikon, Tokyo, Japan) with an epifluorescence package. 10× and 40× water-immersion objectives were used to acquire images of the planar lipid bilayer in Tris buffer solution. Bleaching was achieved with a 100 W high-pressure mercury light source using a contracted field diaphragm, and a 40× objective with a high-resolution air-cooled CCD camera (Photometrics CoolSNAP, Roper Scientific, Planegg, Germany) was used to capture images. MetaMorph software (Molecular Devices, Sunnyvale, CA, USA) was used to collect image stacks to analyze the digitized fluorescence counts of the specified regions. FRAP analysis was performed to extract diffusion coefficients from the temporal fluorescence intensity profiles of selected regions during recovery. The diffusion coefficients were calculated based on the Axelrod method [31].

The normalization is expressed by the fractional form of recovery defined as follows:

(1) $$f(t)=\frac{F(t)-F(0)}{F(t<0)-F(0)}$$

that is fit to a single exponential function for the determination of half-recovery time *τ*_1/2_. The diffusion coefficients can be calculated by the following Equation:

(2) $$D=0.224\cdot \frac{w^{2}}{\tau_{1/2}}$$

where *D* is the fluorophore diffusion constant, *w* is the radius of the bleached area and *τ*_1/2_ is the half recovery time defined by the recovery fraction. The normalized magnitude of fluorescence recovery is usually interpreted as the fraction of the molecular population in the objective that possesses freedom of long-range translation. Therefore, the mobile fraction is determined by:

(3) $$\text{Mobile fraction}=f(t\to \infty )=\frac{F(t\to \infty )-F(0)}{F(t<0)-F(0)}\cdot 100\%$$

where *f* is the normalized recovery defined in Equation (1).

### 2.5. Atomic Force Microscopy

AFM experiments were performed on an XE-100 (Park Systems, Suwon, Korea) in tapping mode with an imaging force of less than 100 pN. Rectangular-shaped Si_3_N_4_ cantilevers with a force constant of *k* = 0.05 N/m and average tip radius of 15 nm (Veeco, Santa Barbara, CA, USA) were used for all experiments. All measurements were performed in Tris buffer [10 mM Tris (pH 7.5), and 150 mM NaCl solution with 1 mM EDTA] using an open liquid cell (Park Systems). The scan line speed was optimized between 0.3 Hz and 2 Hz with a pixel number of 256 × 256, depending on the scan size. Images were recorded in height, amplitude, phase and error modes. All measurements were done on the height images, which were subjected to a first order plane-fitting procedure to compensate for sample tilt. Topographical and grain analyses were performed using XEI 1.5 software (Park Systems).

## 3. Results and Discussion

### 3.1. Size Distribution of Vesicle Suspensions

**Figure 1:**
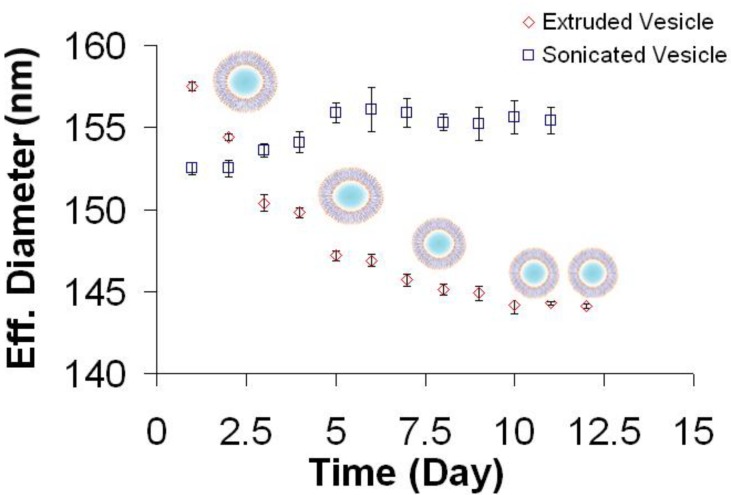
Stability of lipid vesicles produced by different processing techniques. 1-palmitoyl-2-oleoyl-*sn*-glycero-3-phosphocholine (POPC) lipid films dispersed in aqueous buffer and homogenous vesicle suspensions were prepared by pressure extrusion (red diamond) or sonication (blue square). Preparation-dependent effects on vesicle size were monitored by dynamic light scattering as a function of time after processing. Note that the vesicle drawings are merely illustrative and not drawn to scale.

Since vesicles are typically used immediately after preparation for the formation of solid-supported planar bilayers, the relationship between vesicle aging and the interaction of vesicles with solid supports is not systematically studied. In this study, the average size of POPC vesicles was first investigated as a function of preparation method and aging. POPC lipid vesicle suspensions were extruded through 100 nm pore polycarbonate membranes to produce vesicles with an effective diameter of 158 nm, as measured by dynamic light scattering (Figure 1, red diamonds). Over the course of twelve days, the change in effective diameter was monitored. The average size of the vesicles continually decreased until reaching an effective diameter of 144 nm by day 10, which corresponds to an approximately 7% change. Vesicle size then stabilized, and the time range to reach equilibration is consistent with work by Kaler *et al.* for surfactant mixtures [32].

To determine if the observed decrease in effective diameter during the initial period after preparation is a general behavior of extruded vesicles, differently sized vesicles were prepared by extruding samples through 50- or 30-nm pore polycarbonate membranes. Vesicles of all sizes exhibited similar contraction in size upon aging (data not shown), consistent with the notion that uniform vesicles composed of single lipids formed by the extrusion process are, in general, thermodynamically unstable, trapped kinetically in most cases [33]. Mui *et al.* [34,35] have previously shown that the method of extrusion results in production of unilamellar vesicles with non-spherical morphologies, typically oval or sausage-like shapes. The shearing forces induced by the applied pressure during expansion (through the pores) are proposed to create cylindrical bilayer fragments when MLVs pass through the filter pores. Empirical findings suggest that the bilayer area and internal volume of the cylindrical fragments are conserved upon deformation, and that the resulting SUVs are non-spherical (Note that area/volume ratio for cylindrical geometry is larger than a corresponding sphere. As a result, excess membrane perimeter stabilizes flaccid and non-spherical vesicle geometries). These past observations are in good agreement with the light scattering data. The effective diameter determined by DLS assumes a spherical particle shape and calculates the diameter of an equivalent sphere for non-spherical particles. Hence, a non-spherical vesicle would have a greater effective diameter than a spherical vesicle composed of the same amount of lipids. As the result of shape relaxation following extrusion, vesicles become increasingly spherical and the average effective diameter concomitantly decreases until stabilizing, as observed in Figure 1.

In contrast to extruded vesicles, vesicles prepared by sonication maintained a nearly consistent size throughout the course of twelve days, with only a minor 1.5% increase in size from approximately 153 to 155 nm (Figure 1, blue squares). Whereas extruded vesicles show aging effects, sonicated vesicles do not exhibit such behavior and this difference may be due to the mechanism of cavitation induced by ultrasonic irradiation [11]. The resulting shear forces can be quite high and are likely sufficient to promote the formation of tense SUVs. Because in the sonication process, the precursor state consists of large multilamellar aggregates, it seems reasonable that vesicle closure can occur under conditions of area/volume ratios optimal for spherical geometry. In any case, both extrusion and sonication produced monodisperse vesicle samples, thereby indicating that both preparation methods offer excellent control over vesicle size distribution, yielding vesicles with effective diameters of *ca.* 150 nm based on the protocols used in this study. As such, the similar sizes of extruded and sonicated vesicles permit investigation of how vesicle aging affects self-assembly formation of planar lipid bilayers.

### 3.2. Interaction Kinetics of Vesicles with Silicon Oxide

**Figure 2:**
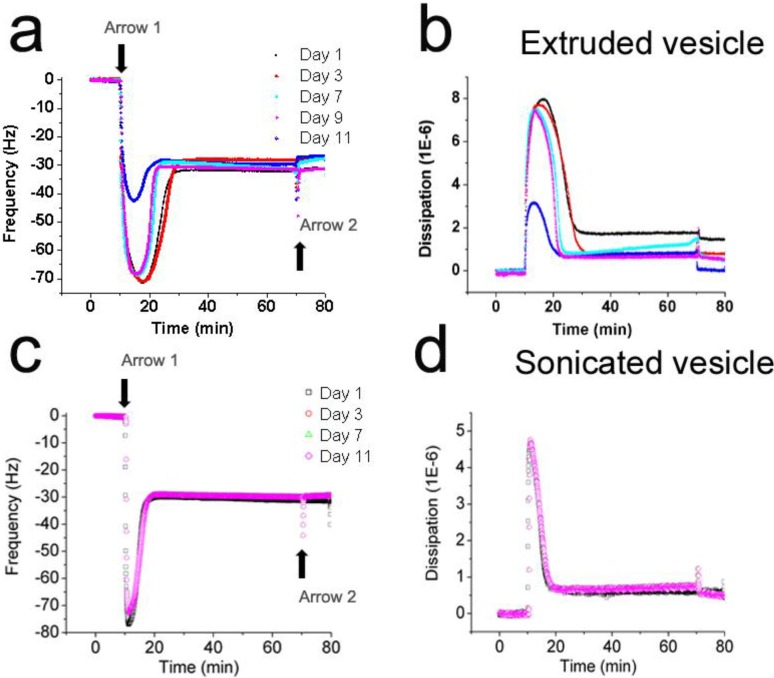
Effects of vesicle aging on the self-assembly of planar lipid bilayers. The interactions of POPC lipid vesicles with silicon oxide substrates were followed by quartz crystal microbalance with dissipation (QCM-D) monitoring as a function of vesicle aging. Normalized changes in (**a**) resonance frequency and (**b**) energy dissipation as a function of time were recorded for lipid vesicles prepared by the extrusion method. Corresponding changes in (**c**) resonance frequency and (**d**) energy dissipation as a function of time were also recorded for lipid vesicles prepared by the sonication method.

Silicon oxide is a hydrophilic substrate that supports the adsorption and rupture of vesicles to promote the self-assembly of a planar lipid bilayer [36]. Thus, the interaction of extruded and sonicated vesicles with a silicon oxide substrate was investigated by the quartz crystal microbalance with dissipation (QCM-D) monitoring technique in order to determine how vesicle aging affects adsorption kinetics and bilayer self-assembly. After the baseline resonance frequency and energy dissipation signals were stabilized in buffer for ten minutes, vesicles were injected (Figure 2, Arrow 1). After planar bilayer self-assembly, a buffer wash was performed to remove nonspecifically bound, remaining intact vesicles, if any (Figure 2, Arrow 2).

Although all samples of extruded vesicles formed planar bilayers, the adsorption kinetics demonstrated age-dependent effects. Three distinct regimes were identified corresponding to the amount of bound vesicles at the critical rupturing concentration and the length of time between initial rupturing and final self-assembly of the planar bilayer. (i) For extruded vesicles between one and three days old, the change in resonance frequency corresponding to adsorbed vesicles reached down to −73 Hz and rupturing kinetics lasted for 9 min; (ii) For vesicles between 7 and 9 days old, similar frequency changes down to −70 Hz were observed, but rupturing kinetics were reduced to 5 min; (iii) In marked contrast, extruded vesicles that were 11 days old elicited a frequency change of only −45 Hz and the rupturing kinetics further decreased to 3 min. Compared to freshly extruded vesicles under identical experimental conditions, the values correspond to ~39% less vesicles necessary to commence rupture and three times quicker rupturing kinetics. Despite these differences, in all cases, the final changes in resonance frequency and energy dissipation values corresponded to complete planar bilayers.

The increased surface coverage required to induce rupture of less aged vesicles demonstrates that more vesicle-vesicle interactions are necessary in these cases. An ~36% decrease in the mass load at the critical rupturing concentration from day 9 to 11 suggests a transition in liposomal properties, and this observation is supported by the DLS data that shows the sample size achieving shape relaxation during the same time period. Additionally, the time at which the critical rupturing concentration is reached, is shorter for more aged vesicles. This observation is consistent with the shape relaxation argument because smaller vesicles would diffuse more quickly to the substrate. Unlike extruded vesicles, the adsorption kinetics of sonicated vesicles did not vary as a function of aging up to 11 days (Figure 2). For all cases, the critical rupturing concentration was reached at approximately −75 Hz and rupturing kinetics lasted for eight minutes until completion of bilayer self-assembly. This uniformity validates the results of the DLS measurements that indicated sonicated vesicles show no aging effects after preparation. While the QCM-D adsorption kinetics indicated formation of a planar bilayer in the various cases, buffer washes performed after the formation process caused mass desorption from planar bilayers formed from extruded vesicles only, and no such changes occurred for bilayers formed from sonicated vesicles (See Figure 2, Arrow 2). Since desorption behavior is typically caused by the loss of weakly adsorbed materials, such as an unstable arrangement of intact vesicles remaining on the substrate among planar bilayer islands in this case, these findings together suggest that sonicated vesicles are more robust for controlled self-assembly of planar lipid bilayers. Building on this work, fluorescence recovery after photobleaching (FRAP) measurements were performed to determine the effects of aging on resulting functional properties of the planar bilayer.

### 3.3. Lateral Mobility of Planar Lipid Bilayer

**Figure 3:**
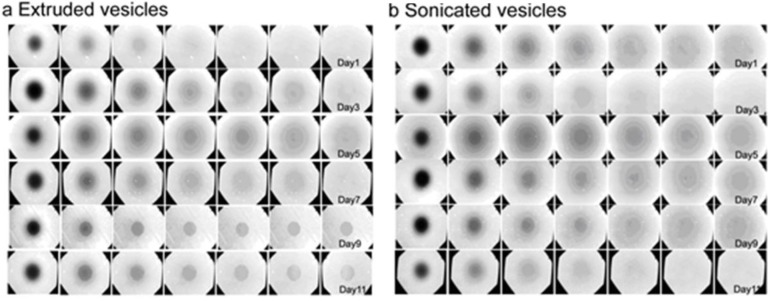
Age-dependent mobility of planar lipid bilayers. Fluorescence recovery after photobleaching (FRAP) experiments were performed on planar lipid bilayers self-assembled from (**a**) extruded or (**b**) sonicated vesicle suspensions of varying age from 1 to 11 days. After photobleaching, fluorescence microscopy images were recorded at 1 min intervals.

The diffusion coefficient of lateral lipid mobility is an important parameter to characterize the fluidity of a planar lipid bilayer. Barriers or other obstructions such as unruptured vesicles can retard mobility, and lower diffusion coefficients and higher fractions of immobile lipids will be observed in these cases [36]. As such, FRAP measurements provide an excellent approach to characterize the integrity and functionality of planar bilayers, and were performed on planar bilayers formed from extruded or sonicated vesicles of varying age. All FRAP experiments were performed simultaneously with QCM-D monitoring in order to directly compare the results (See Figure 2 for QCM-D data and Figure 3 for FRAP data).

As shown in Table 1, planar bilayers formed from freshly sonicated or extruded vesicles have diffusion coefficients of ~2 µm^2^·s^−1^ and mobile fractions of ~80%. These values are in excellent agreement with the findings reported by Lapinski *et al.*, which characterized the mobility of planar bilayers formed from sonicated or extruded vesicles immediately after vesicle preparation [14]. In the present study, planar bilayers formed from sonicated vesicles showed no observable effects of aging, but those formed from extruded vesicles exhibited age-dependent mobility. Bilayers prepared from extruded vesicles up to 7 days of age maintained diffusion coefficients of ~2 µm^2^·s^−1^ and mobile fractions of ~80%, values which are consistent with literature values [37]. However, planar bilayers assembled from extruded vesicles nine days or older had diffusion coefficients of only ~1 µm^2^·s^−1^ and mobile fractions down to ~50%. The timing of this sharp drop in mobility is in good agreement with the transition in adsorption kinetic behavior and further correlates with the size distribution of extruded vesicles achieving shape relaxation.

**Table 1:** Summary values for diffusion coefficient and mobile fraction of planar bilayers as a function of vesicle aging and preparation method.

	Vesicle Aging	Day 1	Day 3	Day 5	Day 7	Day 9	Day 11
**Extrusion**	Diffusion coefficient (μm^2^ s^−1^)	2.12 ± 0.32	2.02 ± 0.22	1.97 ± 0.72	2.01 ± 0.13	0.89 ± 0.64	0.96 ± 0.29
	Mobile fraction (%)	83 ± 9	81 ± 6	79 ± 13	82 ± 9	48 ± 13	53 ± 9
**Sonication**	Diffusion coefficient (μm^2^ s^−1^)	1.89 ± 0.28	1.93 ± 0.35	1.71 ± 0.67	1.98 ± 0.42	1.47 ± 0.62	1.85 ± 0.56
	Mobile fraction (%)	75 ± 5	83 ± 7	73 ± 12	75 ± 15	67 ± 11	79 ± 5

Since planar lipid bilayers are formed through a complex structural transformation that is initiated by the adsorption of intact vesicles onto silicon oxide, it is revealing that planar bilayer properties are dependent on the original method of preparation of the lipid vesicles in solution. Indeed, this preparation-dependence suggests that the lipid re-assembly process whereby intact vesicles transform into a planar bilayer occurs on mesoscopic length scales such that microscopic properties of the lipid membrane are preserved. Furthermore, while the molecular properties of vesicles may be affected by the preparation method, Lapinski *et al.* have shown that rotational and translational diffusion of lipids are similar within either freshly prepared sonicated or extruded vesicles [14]. Taken together with the observation that planar bilayers formed from either freshly prepared sonicated or extruded vesicles have similar self-assembly kinetic profiles and mobilities, it is likely that differences in the aging process for sonicated and extruded vesicles induce structural differences in the lipid assembles which in turn affect self-assembly kinetic and functional properties of planar bilayers. To test this hypothesis, atomic force microscopy (AFM) measurements were performed to investigate the morphology of planar bilayer as a function of vesicle age.

### 3.4. Morphology of Lipid Assemblies on Silicon Oxide

**Figure 4:**
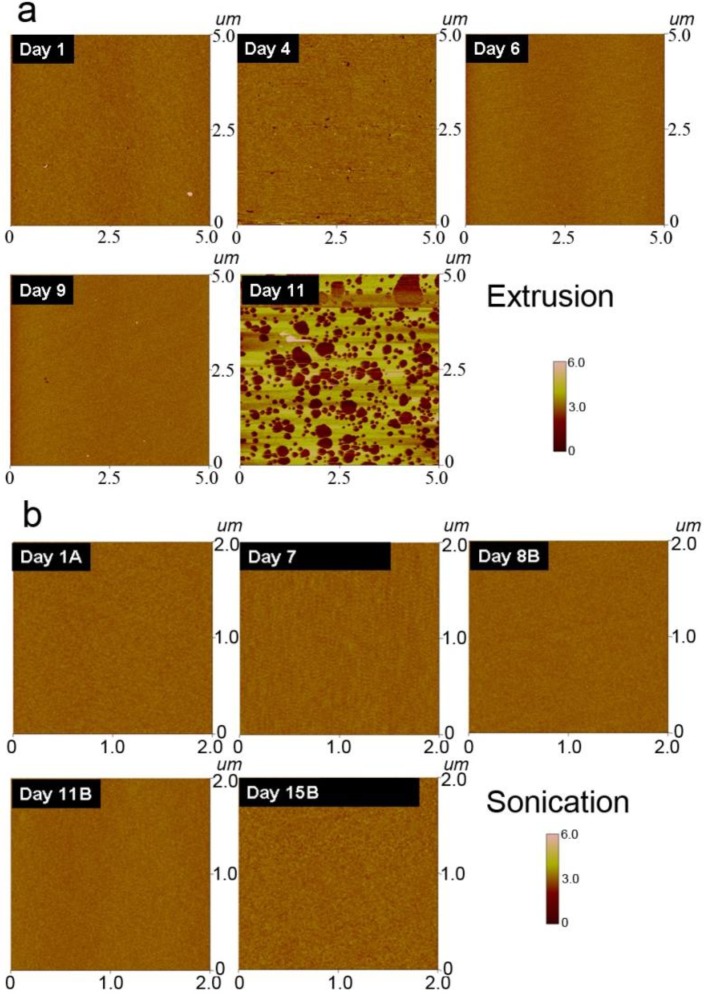
Morphology of planar lipid bilayers as a function of vesicle aging. Atomic force microscopy (AFM) was employed to investigate the morphology of planar bilayers assembled on silicon oxide supports. (**a**) AFM scans presented in height mode were recorded for planar bilayers prepared from extruded lipid vesicles. The scan size is 2 µm × 2 µm; (**b**) Identical scans were also recorded for planar bilayers prepared from sonicated lipid vesicles.

The morphology of planar lipid bilayers reflects the homogeneity and integrity of the platform. Homogenous planar bilayers will display featureless morphologies corresponding to their two-dimensional planar, defect-free structure. By contrast, incompletely formed bilayers have a coarser morphology, including defects such as adsorbed, unruptured vesicles and/or bilayer patches. For these experiments, freshly cleaved mica—another hydrophilic substrate like silicon oxide that promotes the self-assembly of planar lipid bilayers—was used as a solid support to form planar bilayers from aged lipid vesicles initially prepared by either extrusion or sonication. As shown in Figure 4, AFM images were analyzed in height mode in order to distinguish structural differences in the planar bilayer that occurred as a result of vesicle aging.

Planar bilayers assembled from sonicated vesicles exhibited similar morphologies independent of vesicle aging (Figure 4). For vesicles up to 15 days old, all bilayers were uniform and smooth. Likewise, planar bilayers assembled from extruded vesicles up to 9 days old were also homogenous (Figure 4). However, vesicles of 11 days’ age did not completely rupture and instead formed an incompletely assembled planar bilayer. As presented in Figure 4, numerous defects are present among patches of planar bilayer [38], and support the observed decrease in mobility as a function of aging. In particular, the defects can act as barriers to impede lateral diffusivity and in turn significantly decrease the mobile fraction [36]. Another observation to note is that the AFM results did not identify a change in the properties of extruded vesicles until 11 days old whereas FRAP measurements indicated a decrease in mobility for extruded vesicles that were only 9 days old. To explain this difference, it is likely the mechanism which causes vesicle aging occurs gradually. Taken together with differences in the vesicle loading methods for the various experimental techniques and the overall complexity of the vesicle rupture process, the experimental results between techniques are best compared qualitatively based on trends. Indeed, the QCM-D measurements support both the FRAP and AFM experiment by identifying a change in the adsorption kinetics across days 7–11. Collectively, these findings indicate that vesicle aging affects the self-assembly process by preventing the optimal rupturing of extruded vesicles.

Based on the light scattering data, aging may allow extruded vesicles to achieve shape relaxation by osmotic expansion that leads to the self-assembly of incomplete planar bilayers. Interestingly, the aging process results in smaller, spherical vesicles which would seemingly increase the propensity for vesicle rupture. Indeed, the mass load of the critical vesicle concentration that is required to initiate vesicle rupturing decreases as a function of aging. However, in these cases, there are defects remaining, which contribute to reduced lateral lipid diffusion, as measured by the FRAP technique. Based on the shape relaxation argument, it is also important to recall that the kinetic barrier of spontaneous vesicle rupture depends on the tension within the sides of an adsorbed vesicle, which represent the point of highest stress [39]. The process of shape relaxation from an ellipsoid to sphere would reduce the tension on the vesicle sides, and hence increase the barrier to rupture [40]. Therefore, the observed adsorption behavior for aged, extruded vesicles may be the result of shape relaxation influencing both diffusion-limited kinetics and the thermodynamics of the complex rupture process. Additional factors such as size heterogeneity and lipid oxidation may influence vesicle adsorption, although the effect of these factors is likely minor and our experimental observations are most consistent with shape relaxation of extruded vesicles. Taken together, our findings demonstrate that robust self-assembly of homogenous planar lipid bilayers is best achieved with freshly extruded vesicles or alternatively with sonicated vesicles which do not exhibit age-dependent properties within at least 10 days of preparation.

## 4. Conclusions

The combination of biophysical and surface-sensitive analytical techniques employed in this study provides evidence that the process of vesicle aging can affect the quality and functional properties of solid-supported planar lipid bilayers. To establish this finding, DLS measurements first identified that the size distribution of extruded, but not sonicated, vesicles decreases in the days following preparation. Surprisingly, extruded vesicles experience a ~7.5% decrease in average size, presumably the result of shape relaxation following extrusion. By contrast, shear forces induced by cavitation during sonication are likely greater than shear forces induced by applied pressure during extrusion. As such, sonication may cause more drastic disruption of MLVs such that when lipids re-assemble into SUVs, the structures are metastable, albeit kinetically trapped [33]. In any case, QCM-D monitoring detected differences in the adsorption kinetics of sonicated or freshly extruded vesicles *versus* older extruded vesicles. Key parameters affected by vesicle aging include the critical vesicle concentration and time at which vesicle rupturing commences. Subsequent FRAP and AFM studies concluded that these changes in self-assembly kinetics are correlated with a decrease in bilayer mobility as well an increase in bilayer defects. Taken together, it is demonstrated that vesicle aging should be considered as a new parameter for the self-assembly of planar lipid bilayers. While other known experimental parameters such as ionic strength or vesicle size can be directly characterized, the effects of vesicle aging are more subtle and must be understood in order to reproducibly assemble planar lipid bilayers for the increasing number of medical and biotechnology applications.
